# A randomized phase II presurgical trial of weekly low-dose tamoxifen versus raloxifene versus placebo in premenopausal women with estrogen receptor-positive breast cancer

**DOI:** 10.1186/bcr3439

**Published:** 2013-06-20

**Authors:** Davide Serrano, Matteo Lazzeroni, Sara Gandini, Debora Macis, Harriet Johansson, Jennifer Gjerde, Ernst Lien, Irene Feroce, Giancarlo Pruneri, Maria Teresa Sandri, Fabio Bassi, Fabricio Brenelli, Alberto Luini, Massimiliano Cazzaniga, Clara Varricchio, Aliana Guerrieri-Gonzaga, Andrea DeCensi, Bernardo Bonanni

**Affiliations:** 1 Division of Cancer Prevention and Genetics, European Institute of Oncology, via Ripamonti 435, Milan 20141 Italy; 2 Division of Epidemiology and Biostatistics, European Institute of Oncology, via Ripamonti 435, Milan 20141, Italy; 3 Hormone Laboratory, Haukeland University Hospital Institute of Medicine, University of Bergen, Jonas Lies vei 53, N-5021 Bergen, Norway; 4 Division of Pathology, European Institute of Oncology, via Ripamonti 435, Milan 20141, Italy; 5 Unit of Laboratory Medicine, European Institute of Oncology, via Ripamonti 435, Milan 20141, Italy; 6 Division of Senology, European Institute of Oncology, via Ripamonti 435, Milan 20141, Italy; 7 Division of Medical Oncology, E.O. Ospedali Galliera, Mura delle Cappuccine 14, Genova 16128, Italy; 8 University of Milan School of Medicine, via Festa del Perdono 7, 20122 Milan, Italy

**Keywords:** breast cancer, tamoxifen, raloxifene, prevention

## Abstract

**Introduction:**

We previously demonstrated that 1 or 5 mg per day of tamoxifen (T) given for four weeks before surgery reduces Ki-67 in breast cancer (BC) patients to the same extent as the standard 20 mg/d. Given the long half-life of T, a weekly dose (10 mg per week (w)) may be worth testing. Also, raloxifene (R) has shown Ki-67 reduction in postmenopausal patients in a preoperative setting, but data in premenopausal women are limited. We conducted a randomized trial testing T 10 mg/w vs. R 60 mg/d vs. placebo in a presurgical model.

**Methods:**

Out of 204 screened subjects, 57 were not eligible, 22 refused to participate and 125 were included in the study. The participants were all premenopausal women with estrogen receptor-positive BC. They were randomly assigned to either T 10mg/w or R 60 mg/d or placebo for six weeks before surgery. The primary endpoint was tissue change of Ki-67. Secondary endpoints were modulation of estrogen and progesterone receptors and several other circulating biomarkers.

**Results:**

Ki-67 was not significantly modulated by either treatment. In contrast, both selective estrogen receptor modulators (SERMs) significantly modulated circulating IGF-I/IGFBP-3 ratio, cholesterol, fibrinogen and antithrombin III. Estradiol was increased with both SERMs. Within the tamoxifen arm, CYP2D6 polymorphism analysis showed a higher concentration of N-desTamoxifen, one of the tamoxifen metabolites, in subjects with reduced CYP2D6 activity. Moreover, a reduction of Ki-67 and a marked increase of sex hormone-binding globulin (SHBG) were observed in the active phenotype.

**Conclusions:**

A weekly dose of tamoxifen and a standard dose of raloxifene did not inhibit tumor cell proliferation, measured as Ki-67 expression, in premenopausal BC patients. However, in the tamoxifen arm women with an extensive phenotype for CYP2D6 reached a significant Ki-67 modulation.

## Introduction

Presurgical trials offer real 'windows of opportunity' to study tissue biomarkers and their modulation in response to drugs. The effort to study and validate surrogate biomarkers is never wasted since it may lead to better characterization of the tumor response, personalization of adjuvant treatment, and the design of new prevention strategies. The administration of selective estrogen receptor modulators (SERMs) for a period of one to four weeks has been shown to induce a significant antiproliferative effect in estrogen receptor (ER)-positive breast cancers [1,2]. Moreover, we have shown that 5 mg and even 1 mg of tamoxifen maintains a similar antiproliferative effect compared to the standard dose [3]. Recently, the notion has been reinforced that the Ki-67 labeling index (LI) after a short treatment has prognostic implications for disease-free survival and also for overall survival [4,5]

Tamoxifen is the main drug that is able to reduce breast cancer (BC) risk, but its adverse events have so far precluded the uptake as a preventive agent [6]. The Food and Drug Administration (FDA) has approved raloxifene for the same indications as tamoxifen, though only for postmenopausal women. Raloxifene has shown an overall better toxicity profile, but a reduced activity against intraepithelial lesions [7]. Recently, an aromatase inhibitor (exemestane) has also been shown to significantly lower BC risk again in postmenopause [8].

Seeking a better tamoxifen risk/benefit ratio in the prevention setting, we have been studying lower dose tamoxifen activity. Since the drug has a long half-life, a weekly dose might be preferred by healthy women instead of a daily administration. As previously shown, in phase II clinical trials, low-dose tamoxifen (down to 10 mg a week) can favorably modulate circulating biomarkers either in healthy women on hormone replacement therapy, [9], or in women operated on for an ER-positive intraepithelial neoplasia [10].

With this rationale, we performed a randomized presurgical clinical trial with tamoxifen (10 mg a week) or raloxifene (standard dose) versus placebo in premenopausal women with ER-positive early breast cancer. This trial had two main issues to be addressed: does a weekly dose of tamoxifen show an antiproliferative effect on breast cancer relative to placebo? Does raloxifene uphold efficacy and safety in premenopausal women? The primary endpoint was Ki-67 LI modulation. Several other secondary endpoints were measured including circulating breast cancer risk biomarkers, such as insulin-like growth factor (IGF) system and hormone levels; cardiocirculatory biomarkers such as fibrinogen, antithrombin III, C-reactive protein (CRP) and cholesterol; bone metabolism biomarkers, in particular C-telopeptide (CTX) and osteocalcin.

## Material and methods

### Study design

This monoinstitutional study was conducted from 2004 to 2009. It is a three-arm randomized double-blind clinical trial in premenopausal women with confirmed hormone-responsive breast cancer. The three arms are: raloxifene 60 mg/day versus tamoxifen 10 mg/week versus placebo in a 2:2:1 ratio for six weeks. By the time the study was implemented, at the European Institute of Oncology, there was a waiting time for early-stage breast cancer surgery of approximately six to eight weeks. The study (IEO number 162, register number ISRCTN86894592) and all amendments during its conduct were approved by the Institutional Review Board (the European Institute of Oncology Ethical Committee), and all subjects gave their written informed consent. Tumor biopsy and blood samples were taken to check eligibility criteria and were stored for endpoint analysis before the systemic treatment began. Blood samples and breast tissue were taken again at surgery. One hundred and twenty-five premenopausal patients were included in the study. The primary endpoint was Ki-67 LI tissue change. Secondary endpoints were the assessment of the changes in ER and progesterone receptor (PgR) expression, and other circulating biomarkers.

Inclusion criteria were: female, aged 18 years or older; performance status = 0 (SWOG); histologically confirmed ER+ primary breast cancer; stage T_1-2_, N_0-1_, M_0 _or women with larger tumors refusing neoadjuvant treatment before surgery; no previous breast cancer treatment; written informed consent.

Exclusion criteria were: patients eligible for chemotherapy and/or endocrine neoadjuvant therapy; evidence of previous superficial or deep venous thrombosis or other major thromboembolic events (pulmonary embolism, stroke, and so on); current anticoagulant therapy; moderate to severe alteration in hematologic profile, hemostasis, renal and hepatic metabolism; clinically active peptic ulcer or gastroenteric disease; severe retinal disease; severe endometriosis (grade III to IV) or other proliferative disorders of the endometrium; clinically active neurologic or psychiatric disease; other medical contraindications according to the investigator; other coexisting malignancies or malignancies diagnosed within the last five years with the exception of basal cell carcinoma or *in situ *cervical cancer; pregnancy or current breast-feeding (women of child-bearing potential must have had a negative pregnancy test within seven days before the start of the study treatment).

Treatment compliance was monitored by pill count, the calendar filled out by the patient, and drug plasma concentrations.

### Sampling of biological specimens

Fasting blood samples were taken preferably between 8 and 10 a.m. at baseline and on the day before surgery. EDTA blood samples were collected and stored unprocessed in aliquots in the freezer at -80°C until DNA extraction was performed. Serum and plasma were separated by 10 minute centrifugation at 1350 × g and stored as aliquots at -80°C until assayed for biomarkers.

Prior to randomization two sequential core biopsies of the primary tumor were performed percutaneously with a 14-gauge needle after local anesthetic was administered. The location of the biopsy was chosen on the basis of the involved area of the breast and the consequent surgical incision. At the time of surgery, a representative sample of the cancerous excision tissue was obtained, together with a specimen of the contiguous normal tissue.

### Assay methods

#### Circulating biomarkers

IGF-I and insulin-like growth factor-binding protein 3 (IGFBP-3) levels were determined on serum samples by a solid-phase enzyme-labeled chemiluminescent immunometric assay on the Immulite 2000 Siemens analyzer (Siemens AG, Erlangen, Germany). The analytical sensitivity of the test was 20 ng/mL for IGF-I and 0.1 μg/mL for IGFBP-3. Total cholesterol, high-density lipoprotein (HDL) cholesterol, and triglycerides serum levels were determined by enzymatic colorimetric methods with a Cobas Integra (Roche Diagnostics S.p.A., Monza, Italy), a fully mechanized multichannel analyzer for routine clinical chemistry purposes. Methods were performed according to specific instructions. Low-density lipoprotein (LDL) cholesterol was obtained according to the Friedewald formula (LDL cholesterol = total cholesterol - HDL cholesterol - (triglycerides/5)) [11]. Plasma fibrinogen and antithrombin III were assayed on plasma citrate samples using the ACL Elite Pro Analyzer (Instrumentation Laboratory, Bedford, MA, USA). In this assay, clot detection is performed using a photo-optical technology. Serum concentration of sex hormone-binding globulin (SHBG was measured by a solid-phase, two-site chemiluminescent immunometric assay on the Immulite 2000 Siemens automated analyzer. The sensitivity of the assay was 0.02 nm//L. High-sensitivity C-reactive protein (hs-CRP) was determined by the enzymatic turbidimetric method on the Cobas Integra analyzer. The sensitivity of the test was 0.1 mg/L. Serum CTX and osteocalcin, estradiol and testosterone were determined by an electrochemiluminescent immunometric assay (Roche Diagnostics S.p.A., Monza, Italy) designed for the Cobas e411 automated analyzer. The sensitivity of the assays was 0.01 ng/mL and 0.50 ng/mL for CTX and osteocalcin, and 5 pg/mL and 0.025 ng/mL for estradiol and testosterone, respectively.

With the exception of the lipid profile, fibrinogen and antithrombin III, which were determined on fresh specimens, pre- and posttreatment serum samples obtained from each subject were simultaneously assayed on frozen samples to eliminate the effects of interassay variation. In these assays, in addition to the specific control samples that come with the assay kits, an in-house pooled control sample of serum obtained from healthy donors was used to monitor the coefficient of variation between assays.

Tamoxifen citrate and 4-hydroxytamoxifen (4-OHtam) were purchased from Sigma-Aldrich (Steinheim, Germany), the internal standard deuterated 5-tamoxifen (D5tam) and tamoxifen-N-oxide (tamNox) from Beta Chem Inc. (Kansas, USA), and 4-hydroxy-N-desmethyltamoxifen (endoxifen) from Sintef Materials and Chemistry (Oslo, Norway). N-desmethyltamoxifen (N-desTam) and N-desdimethyltamoxifen (N-desDTam) were gifts from the Pharmaceuticals Division of Imperial Chemical Industries PLC (Macclesfield, UK). To determine the concentrations of tamoxifen and its metabolites, a high-pressure liquid chromatography-tandem mass spectrometry system was used [12,13]. The assay was modified to improve the sensitivity by changing the API 2000/Qtrap mass spectrometry system from Applied Biosystems (AB MDS Sciex, Concord, Canada) to the API 4000, equipped with TurboIonSpray.

#### Pathology and immunohistochemistry

Biopsy and surgical specimens were fixed in 10% neutral-buffered formalin for 6 to 8 hours before being embedded in paraffin. Sections (4 micron thick) were cut and stained with hematoxylin and eosin. Consecutive serial sections were used for immunohistochemical determinations. Expressions of ER, PgR, Ki-67, human epidermal growth factor receptor 2 (Her2)/neu were determined by immunohistochemistry (IHC). Briefly, dewaxed tumor sections were pretreated with 3% hydrogen peroxide for 5 minutes to block endogenous peroxidase activity and then treated with a solution of 0.001 M EDTA (pH 8.0) at 99°C for 20 minutes to retrieve antigens. The tumor sections were then incubated with primary mouse monoclonal antibodies to ER (clone 1D5, 1:100 dilution), PgR (clone 1A6, 1:800 dilution), Ki-67 (clone Mib-1, 1:200 dilution), or with rabbit polyclonal antibody to the Her2/neu protein (1:3200 dilution). Tumor subtypes were classified by IHC into four categories according to the 2011 St. Gallen criteria [14]: luminal A, ER- or PgR-positive and Ki-67 <14%; luminal B-, ER- or PgR-positive, HER2-positive and Ki-67 ≥14%; HER2-positive, HER2 3+ or fluorescence *in situ *hybridization (FISH) amplified and ER- and PgR-negative; triple negative, all three receptors negative.

#### Genotype analysis

Genomic DNA was extracted from whole blood specimens with a QIAamp DNA blood kit (Qiagen, Valencia, CA, USA). The INFINITI analyzer (AutoGenomics, Carlsbad, CA, USA) was employed for CYP2D6 genotyping, according to the manufacturer's protocol. The test screens for the following CYP2D6 allele variants: *2 (2850C>T) (normal activity); *3 (2549delA), *4 (1846G>A), *5 (CYP2D6 deleted), *6 (1707 delT), (no activity); *7 (2935A>C); *8 (1758G>T); *9 (2615_2617delAAG); *10 (100C>T); *12 (124G>A); *14 (1758G>A); *17 (1023C>T); *29 (1659G>A); *41A (2988G>A) (reduced activity), and *XN (multiple CYP2D6). We classified the resulting phenotype as poor metabolizers (PM) when there were nonfunctional variants on both alleles; intermediate metabolizers (IM) if there was at least a slow or nonfunctional allele; extensive metabolizers (EM) when both alleles were normal; ultrarapid metabolizers (UM) when normal alleles were amplified. For the analysis we grouped EM + UM as active, and IM + PM as reduced activity.

### Statistical analysis

In this phase II presurgical trial, we tested the difference in the percentage change reduction in Ki-67 LI by treatment. The primary endpoint of the study was to assess drug efficacy, measured as Ki-67 LI percentage change in patients treated for six weeks before surgery.

All randomized subjects were evaluated according to the intention-to-treat approach. The statistical analysis of the primary and secondary endpoints was the comparison between the median change from baseline to surgery and between the treatment arms. Ki-67 values were log-transformed to reach normality; this procedure was adopted for other variables when necessary.

Two orthogonal contrasts were used to compare biomarker changes among treatment groups: any treatment versus placebo and tamoxifen versus raloxifene. These contrasts were specified *a priori *and are consistent with the aims of the study.

The effect of potential covariates, such as age, body mass index (BMI), tumor grade and size, and baseline levels of circulating biomarkers, together with their interactions with treatment were investigated through the ANCOVA analysis.

As exploratory investigations, we evaluated the effect of CYP2D6 phenotype on the variations of metabolites, Ki-67 expression and other biomarkers, as a factor in the ANOVA models, using the available data.

Median values, interquartile ranges and results from nonparametric Wilcoxon tests were presented to investigate differences among the treatment arms of subjects' demographics and tumor characteristics at baseline, when evaluated as continuous variables. Frequencies, chi-squared or Fisher's exact tests were used to present and analyze the association between categorical variables.

All statistical tests were two-sided. Analyses were performed using SAS statistical software (version 9.0, SAS Institute Inc, Cary, NC, USA).

## Results

**Figure 1:**
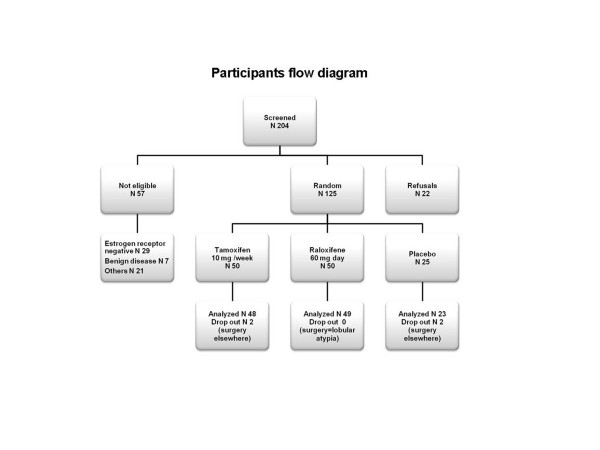
Consort statement

From February 2004 to November 2009, a total of 204 women were registered and assessed to determine their eligibility for the study (Figure 1). Of those women, 57 were not eligible, a further 22 women refused to participate in the study, and 125 women were randomized. Subject demographics and tumor characteristics at baseline are shown in Table 1. There were no statistically significant differences among the three arms. Treatment compliance was over 80% by pill count. Drug plasma levels were measured and confirmed an adequate drug intake; raloxifene levels ranged from 28 to 65.5 ng/mL. Tamoxifen plasma concentration ranged from 7.68 to 11.7 ng/mL and the endoxifen range was 2.52 to 4.1 ng/mL.

**Table 1:** Subjects' clinical characteristics at baseline.

	Placebo (25)	Raloxifene (50)	Tamoxifen (50)
Age (Lower-upper quartile)	44 (42-47)	46 (42-49)	45 (41-49)

Age at menarche (Lower-upper quartile)	13 (12-13)	12 (12-13)	12 (11-14)

Parity	1	2	2

Family history^1^ BC°- OC°°	5 - 1	15 - 1	11 - 3

Body mass index (Lower-upper quartile)	22.9 (21.7-26.2)	22.2 (20.5-24.6)	22.2 (20.3-25.2)

Smoking Never/former/current/ missing	14/3/8/0	28/10/11/1	24/5/21/0

Tumor size mm (Lower-upper quartile)	22.0 (15-30)	22.5 (15.0-28.5)	21.5 (15.0-27.5)

Tumor grade 1/2/3/unknown^2^	7/7/5/6	3/23/7/17	3/22/8/17

HER2 overexpression % No/yes	96/4	98/2	94/6

Luminal subtype % A/B HER2 neg/B HER2 pos	36/60/4	20/78/2	27/67/3

^1^First or second degree relatives with °breast cancer (BC) or °°ovarian cancer (OC); ^2^not graded by the pathologist.

### 

#### Endpoint tissue biomarkers

A weekly dose of 10 mg of tamoxifen did not show a significant antiproliferative effect in premenopausal women. Likewise, raloxifene had a similar null effect on Ki-67 LI, the mean change being 0 in the three arms. The *P *value was 0.78 and 0.76 for treatment vs. placebo and tamoxifen vs. raloxifene, respectively (Table 2). Also ER (data not shown) and PgR (Table 2) were not significantly modulated by either drugs. For PgR, the median change was 0 in the placebo and raloxifene arm, whereas the tamoxifen group was 4.5 (*P *value 0.26 and 0.10 for treatment vs. placebo and tamoxifen vs. raloxifene, respectively).

**Table 2:** Median change of the Ki-67 labeling index and progesterone receptors

Variable	Arm	Baseline	Surgery		
		
		Median IQR	Median IQR	Median change	Contrast *P*
		
Ki-67	P	16 (9 to 22)	15 (10 to 23)	0 (-4 to 3)	T vs. R *P = *0.76
	T	18 (14 to 28)	19.5 (12.5 to 26.5)	0 (-5 to 4)	T + R vs. P *P = *0.78
	R	21.5 (15 to 27)	21 (15 to 25)	0 (-5 to 4)	
**PgR**	P	80 (65 to 95)	90 (60 to 95)	0 (0 to10)	T vs. R *P = *0.10
	T	65 (10 to 90)	65 (22.5 to 92.5)	4.5 (-0.5 to 12.5)	T + R vs. P *P = *0.26
	R	80 (45 to 90)	70 (20 to 90)	0 (-5 to 5)	

**ER**	P	90 (80 to 95)	90 (80 to 95)	0 (0 to 1)	T vs. R *P = *0.99
	T	90 (80 to 90)	90 (80 to 95)	1 (0 to 5)	T + R vs. P *P = *0.82
	R	90 (90 to 95)	90 (85 to 95)	0 (0 to 1)	

IQR, interquartile range; P, placebo; T, tamoxifen 10 mg/week; R, raloxifene 60 mg/day; PgR, progesterone receptor; ER, estrogen receptor.

#### Circulating biomarkers

Both drugs showed a significant biologic effect on circulating biomarkers. As BC risk biomarkers, IGF-1, IGFBP-3, SHBG, estradiol, and testosterone were assessed (see Table 3). Both SERMs significantly modulated circulating IGF-1/IGFBP-3 ratio, the median change was -0.01 in the tamoxifen group, -0.03 in the raloxifene arm, and 0 in the placebo arm (*P *value for treatment vs. placebo 0.008). Estradiol mean change was 27 and 29.5 for raloxifene and tamoxifen, respectively (treatment vs. placebo *P *= 0.053).

**Table 3:** Median change (interquartile range) of circulating biomarkers

			Baseline	Six weeks	Change		
					
Variable	**Treat**.	N Obs	Median IQR	Median IQR	Median IQR	Contrast^1^	***P***
IGF1 ng/mL	P	25	146 (117 to 177)	151 (105 to 205)	-0.5 (-14.5 to 12)	T vs. R	0.87
	R	50	134.5 (113 to 174)	126 (104 to 157)	-10.1 (-30.4 to 13)	T + R vs. P	0.15
	T	50	137.5 (113 to 170)	137 (92.7 to 168)	-13 (-27 to 11)		

IGFBP-3 µg/mL	P	25	4.16 (3.75 to 4.76)	4.42 (3.79 to 4.84)	-0.09 (-0.29 to 0.22)	T vs. R	**0.05**
	R	50	4.22 (3.84 to 4.71)	4.58 (4.07 to 5.11)	0.41 (0 to 0.69)	T + R vs. P	0.22
	T	50	4.28 (3.72 to 4.73)	4.42 (3.87 to 4.78)	0.02 (-0.25 to 0.37)		

IGF1/BP3 ratio	P	25	0.18 (0.16 to 0.21)	0.18 (0.15 to 0.24)	0 (-0.03 to 0.02)	T vs. R	0.41
	R	50	0.17 (0.15 to 0.21)	0.16 (0.12 to 0.17)	-0.03 (-0.05 to 0)	T + R vs. P	**0.01**
	T	50	0.18 (0.15 to 0.2)	0.17 (0.13 to 0.19)	-0.01 (-0.04 to 0)		

SHBG nmol/L	P	25	65.9 (39 to 94)	53.5 (36 to 86)	-7.7 (-22 to 3.4)	T vs. R	0.92
	R	50	63.45 (43.3 to 96.4)	78.1 (50.7 to 101)	7.7 (-7.3 to 18.5)	T + R vs. P	0.32
	T	50	64.3 (47.8 to 80.4)	69.6 (51.7 to 86.8)	2.2 (-7.1 to 21.9)		

Testosterone	P	25	0.26 (0.17 to 0.34)	0.28 (0.16 to 0.35)	0.02 (-0.08 to 0.07)	T vs. R	0.90
ng/mL	R	50	0.23 (0.16 to 0.32)	0.27 (0.18 to 0.36)	0.03 (-0.03 to 0.09)	T + R vs. P	0.10
	T	50	0.23 (0.19 to 0.32)	0.27 (0.22 to 0.34)	0.02 (-0.02 to 0.09)		

Estradiol ng/mL	P	25	120.3 (51.4 to 167.4)	104.35 (45.1 to 179.4)	-9.6 (-99.6 to 91)	T vs. R	0.45
	R	50	94.35 (41.9 to 147.4)	124.7 (65.5 to 209)	27 (-62.9 to 159.08)	T + R vs. P	**0.05**
	T	50	103.25 (61.98 to 165.5)	160.7 (70.86 to 306.4)	29.5 (-37.3 to 179.8)		

IQR, interquartile range; IGF, insulin-like growth factor; P, placebo; R, raloxifene 60 mg/day; T, tamoxifen 10 mg/week; IGFBP-3, insulin-like growth factor-binding protein 3; SHBG, sex hormone-binding globulin.

*Contrast are between tamoxifen arm versus Raloxifene are and either treatment versus placebo.

No difference was observed between treatment and placebo for SHBG, IGFBP-3, IGF-1 and testosterone. There was a difference between the two SERMs for IGFBP-3, with a greater increment of the binding protein with raloxifene, mean change 0.41 vs. 0.02 (tamoxifen vs. raloxifene *P *= 0.046).

The following cardiovascular risk biomarkers were analyzed: cholesterol, fibrinogen, antithrombin III, and CRP (see Table 4). With the exception of CRP, all the other biomarkers were significantly modulated by treatment compared to placebo. Cholesterol median change was -12 and -7, antithrombin III was -7 and -8, fibrinogen -2 and -29.5 for tamoxifen and raloxifene, respectively. The *P *value for treatment vs. placebo for cholesterol, fibrinogen, and antithrombin III was *P *<0.0001, *P *= 0.017 and *P *= 0.009, respectively. Fibrinogen showed a difference also between the two SERMs, with a greater reduction by raloxifene compared to tamoxifen (*P *= 0.017).

**Table 4:** Median change of circulating biomarkers.

			Baseline	Six weeks	Change		
					
Variable	Treat.	N Obs	Median IQR	Median IQR	Median IQR	**Contrast**^1^	***P***
Fibrinogen	P	25	312.0 (273.0 to 340.0)	333.5 (276.0 to 354.0)	4.5 (-19.0 to 24.0)	T vs. R	**0.02**
mg/dL	R	50	301.0 (254.0 to 334.0)	260.0 (230.0 to 288.0)	-29.5 (-61.0 to -5.0)	T + R vs. P	**0.01**
	T	50	286.0 (247.0 to 319.0)	285.0 (243.0 to 312.0)	-2.0 (-20.0 to 12.0)		

Antithrombin	P	25	102.0 (98.0 to 109.0)	99.0 (93.0 to 104.0)	-1.0 (-5.0 to 4.0)	T vs. R	0.75
%	R	50	103.0 (98.0 to 108.0)	94.0 (90.0 to 101.0)	-8.0 (-11.0 to -1.0	T + R vs. P	**0.02**
	T	50	106.0 (98.0 to 114.0)	100.0 (92.0 to 106.0)	-7.0 (-16.0 to -1.0)		

CRP	P	25	0.6 (0.3 to 2.8)	0.8 (0.3 to 3.3)	0.0 (-0.1 to 0.6)	T vs. R	0.12
mg/dL	R	50	1.0 (0.5 to 1.9)	0.6 (0.4 to 1.3)	-0.3 (-0.7 to 0.1)	T + R vs. P	0.21
	T	50	0.7 (0.4 to1.2)	0.8 (0.4 to 1.3)	0.1 (-0.3 to 0.3)		

Cholesterol	P	25	199.0 (185.0 to 224.0)	225.5 (197.0 to 244.0)	13.5 (1.0 to 33.0)	T vs. R	0.30
mg/dL	R	50	199.5 (179.0 to 222.0)	192.0 (168.0 to 215.0)	-7.0 (-23.0 to 5.0)	T + R vs. P	**<.0001**
	T	50	201.5 (180.0 to 220.0)	188.5 (171.0 to 200.0)	-12.0 (-18.0 to 0.0)		

CTX ng/mL	P	25	0.3 (0.2 to 0.4)	0.3 (0.2 to 0.3)	0.0 (-0.1 to 0.0)	T vs. R	**0.05**
	R	50	0.3 (0.2 to 0.4)	0.3 (0.2 to 0.4)	0.0 (0.0 to 0.1)	T + R vs. P	0.58
	T	50	0.3 (0.2 to 0.4)	0.3 (0.2 to 0.4)	0.0 (-0.1 to 0.0)		

Osteocalcin	P	25	16.9 (13.4 to20.3)	16.5 (13.1 to 22.5)	-0.5 (-1.9 to 2.2)	T vs. R	0.39
ng/mL	R	50	17.9 (13.8 to 22.5)	16.8 (13.4 to 20.8)	-1.7 (-3.5 to 1.1)	T + R vs. P	0.32
	T	50	16.0 (61.98 to 165.5)	15.3 (12.5 to 20.2)	-0.7 (-2.0 to 1.0)		

^1^Change adjusted for baseline. CRP, C-reactive protein; CTX, C-telopeptide; IQR, interquartile range; P, placebo; R, raloxifene 60 mg/day; T, tamoxifen 10 mg/week.

Table 4 also shows CTX and osteocalcin biomarkers to investigate bone metabolism. Overall the treatment did not lead to any significant modulation of the two biomarkers. For CTX, we observed a median change of -0.03 in the tamoxifen group and 0.02 in the raloxifene arm, with a borderline significant difference between them (*P *value 0.051 tamoxifen vs. raloxifene). Osteocalcin was unchanged in all three arms.

#### Genotype, phenotype and tamoxifen metabolites

**Figure 2:**
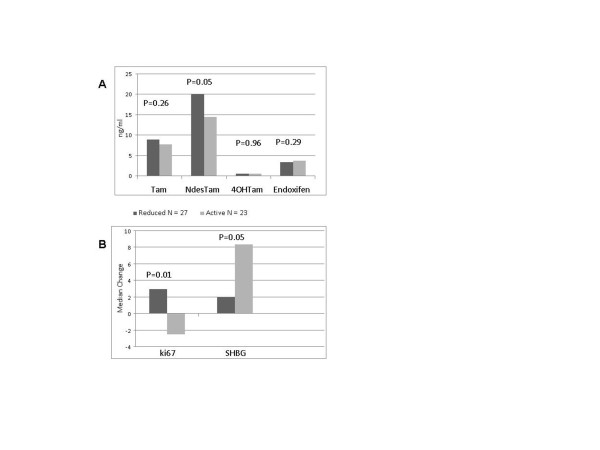
**CYP2D6 phenotype, tamoxifen metabolites and biomarkers modulation**. **(a) **Tamoxifen and its major metabolite plasma level based on CYP2D6 phenotypes. A significant accumulation of N-desmethyltamoxifen is noticed in the reduced activity phenotype. **(b) **Ki-67 labeling index (LI) and sex hormone-binding globulin (SHBG) modulation based on the different phenotypes. A significant decrease of Ki-67 LI and an increase of SHBG can be seen in the active group.

Genotype analysis for CYP2D6 was performed in all the subjects. The frequencies were respectively: tamoxifen arm 40% of EM, 48% of IM, 6% of PM, and 6% of UM; raloxifene arm 32% of EM, 64% of IM, 4% of PM; placebo arm 36% of EM, 56% of IM, 8% of PM. Tamoxifen and its metabolite concentrations were measured, and the correlation with the phenotype was evaluated. The phenotypes were grouped in two classes: active (EM + UM) and with a reduced activity (IM + PM). As shown in Figure 2a, a significant accumulation of N-desTam was present in the reduced activity group, the mean plasma level of N-desTam was 19.9 in the reduced group and 14.4 ng/mL in the active one (*P *= .05). Endoxifen and 4-OHtam were not statistically different in the two phenotype groups. Figure 2b shows the correlation between phenotype and biomarker modulations. We selected those which are more directly correlated with breast cancer risk. Interestingly, the active phenotype showed a significantly different modulation in Ki-67 with a median change of -2.5 in the active group and +2.5 in the reduced one, while no difference in ER or PgR expression was determined by the phenotype (data not shown). Among the circulating biomarkers, SHBG showed a significant difference in the active group as compared to the reduced one (median change 8.35 and 2 *P *= 0.05), whereas estradiol and IGF-1/BP3 did not show any changes between the groups (data not shown).

In order to confirm that these observations are due to tamoxifen and phenotype interaction we also analyzed the effect of the phenotype of the other two arms. Table 5 shows the lack of Ki-67 modulation in the no tamoxifen treated subjects by phenotype, and the significant tamoxifen - phenotype interaction (*P *= 0.01).

**Table 5:** Tamoxifen versus no tamoxifen and phenotype interaction on Ki-67 modulation.

Variable	Reduced		Active	
	Tamoxifen Median (Low/upper quartile)	No tamoxifen Median (Low/upper quartile)	Tamoxifen Median (Low/upper quartile)	No tamoxifen Median (Low/upper quartile)
	
Ki-67 change	2.5 (-2/7)	0 (-5/2)	-2.5 (-10/0)	0 (-1/4)

## Discussion

The efficacy of tamoxifen in lowering breast cancer risk has been confirmed in long-term follow-up of the main chemoprevention trials [15]. In spite of these very strong data, the use of tamoxifen in the clinical practice for breast cancer prevention in high-risk women has shown little increase. For postmenopausal women, raloxifene showed substantial breast cancer risk reduction in the STAR study [7], although inferior to tamoxifen during follow-up [16]. As a preventive strategy, raloxifene may have more appealing characteristics compared to tamoxifen, since it has a better safety profile and is not commercially labeled as an anticancer drug; both are important aspects to consider in preventive care. It would be very interesting to study and to extend the raloxifene indications to premenopausal women.

The data obtained by presurgical studies are useful tools not only in studying drug activity, but also in evaluating prognostic and predictive factors. This allows improved tailoring of treatment both in the adjuvant and the preventive setting [4,5,17].

In the preventive setting, our group is striving to improve the risk-benefit profile of tamoxifen, investigating the minimal effective dose.

The main question of this study was whether 10 mg per week of tamoxifen and standard dose raloxifene can lower Ki-67 LI after six weeks of treatment in premenopausal women operated on for ER-positive breast cancer. The results here reported showed no effect of the two drugs on Ki-67 LI. These data differ from a previous study where the effect of low-dose tamoxifen (down to 1 mg/day) reduced Ki-67 LI in women with breast cancer [3]. Also, PgR expression was not modulated by the treatment; these data are consistent with a previous study by Clarke *et al*. with tamoxifen at standard dose [1]. Two aspects must be mentioned: first, the participants of the previous study were mostly postmenopausal. Second, although the cumulative dose of 10 mg per week is greater than 1 mg per day, tamoxifen having an approximate eight-day half-life [18], the plasma concentration of 10 mg per week was lower compared to the concentration of 1 mg per day measured in the earlier study [3]. On the other hand, the tamoxifen plasma level reported here is consistent with a previous study with the same schedule. In that study, the drug was measured at three, six, and twelve months [10].

According to our previous data [19], at this drug concentration the influence of CYP2D6 genotype shows a significant difference only for N-desTam, the endoxifen substrate, and not for endoxifen itself. Significantly higher levels of the substrate were shown in the reduced enzyme activity subjects. It was interesting to observe that, although endoxifen concentration was not different in the two phenotypes, the extensive metabolizers lowered Ki-67 LI and greatly increased SHBG. Since estradiol was not differently modulated one can speculate that the extensive phenotype may have a greater antagonistic activity compared with the reduced one. Although CYP2D6 polymorphisms *per se *showed conflicting results at a clinical level [20-23], they may be relevant for specific tamoxifen biological activities and play a role in a broader picture as suggested by Dunn *et al. *[24].

Similarly to tamoxifen, raloxifene has also showed a Ki-67 LI reduction in a presurgical study; yet again, that was in postmenopausal subjects only [2]. Other phase II clinical trials have studied raloxifene in premenopausal women. A first report showed a reduction in fibroglandular tissue volume detected by magnetic resonance imaging (MRI), but the mammographic density was unchanged, after one and two years of treatment [25]. The same group showed that raloxifene significantly increased plasma level of estradiol and SHBG [26]. Our results are consistent with these data, moreover we have shown a reduction of the IGF-1-BP3 ratio.

Little is known about raloxifene bioavailability: the plasma concentration detected in our patients is relatively high considering the peak reached with a single-dose administration [27].

SERMs, tamoxifen in particular, have a quite complex balance between agonist and antagonist effects. This is influenced by several variables, such as dosage, target tissue, expression coactivators, and hormonal milieu [28-31]. Differences in menopausal status and dose schedule in the population of the study may explain our different results as compared to the previous trials. In the NSABP-P1, tamoxifen was shown to be slightly less effective in premenopausal subjects as compared to postmenopausal women [32].

Although there was no effect on Ki-67 LI, a biological drug activity is shown by the modulation of the circulating biomarkers. On average, the treatment increased SHBG and estradiol, and decreased the IGF-1/IGFBP-3 ratio as compared to placebo. Cholesterol, antithrombin III, and fibrinogen were significantly decreased by treatment. On the other hand, no differences were observed on testosterone, CRP, osteocalcin, and C-telopeptide. Although the circulating biomarkers were modulated by treatment, no correlation with clinical outcome can be drawn. No major differences between drugs were noticed on any surrogate biomarkers, except for a slight increase of IGPBP-3 and a substantial reduction of fibrinogen with raloxifene over tamoxifen. These findings may suggest that the bioactivity of low-dose tamoxifen is comparable to standard-dose raloxifene.

Lacking any effect on primary endpoint, that is Ki-67 LI reduction, it is difficult to support a definite preventive role of a weekly dose of tamoxifen or raloxifene in premenopause. However, the unchanged Ki-67 LI does not necessarily means a lack of preventive activity. Our endpoint, cancer cell proliferation, is focused on a final step along the pathological process. SERMs activity is certainly broader than merely a cytostatic or cytotoxic effect. Although both drugs modulate sex hormones, the IGF system and other circulating biomarkers, none of these have been validated as surrogate biomarkers for cancer prevention. Tamoxifen, at such low doses, most probably cannot successfully compete with ERs in the presence of premenopausal estradiol levels. Considering our previous and current studies, we are now focusing, in the prevention setting, on tamoxifen at 5 mg per day. Raloxifene, considering our data together with those of other studies, may have biological activity in premenopausal women, but its possible role in breast cancer prevention has to be still confirmed.

## Conclusions

Tumor cell proliferation, in premenopausal breast cancer patients, was not reduced by a weekly low dose of tamoxifen and standard dose of raloxifene. Yet, at a biological level, both drugs did show some activity, modulating some circulating biomarkers. Interestingly, subjects' phenotype for CYP2D6 differentially modulated the tamoxifen effect on Ki-67 and SHBG, showing an enhancement of activity in the extensive phenotype subjects.

## Abbreviations

4-OHtam: 4-hydroxytamoxifen; BC: breast cancer; BMI: body mass index; CRP: C-reactive protein; CTX: C-telopeptide; D5tam: deuterated 5-tamoxifen; EM: extensive metabolizers; ER: estrogen receptor; FISH: fluorescence *in situ *hybridization; HER2: human epidermal growth factor receptor 2; HDL: high-density lipoprotein; IGF: insulin-like growth factor; IGFBP: insulin-like growth factor-binding protein; IHC: immunohistochemistry; IM: intermediate metabolizers; LDL: low-density lipoprotein; LI: labeling index; MRI: magnetic resonance imaging; N-desDTam: N-desdimethyltamoxifen; N-desTam: N-desmethyltamoxifen; OC: ovarian cancer; P: placebo; PgR: progesterone receptor; PM: poor metabolizers; R: raloxifene; SERMs: selective estrogen receptor modulators; SHBG: sex hormone-binding globulin; T: tamoxifen; tamNox: tamoxifen-N-oxide; UM: ultrarapid metabolizers; w: week.

## Competing interests

The authors declare they have no competing interests.

## Authors' contributions

DS, AGG, AD and BB designed the study, drafted the manuscript and revised it for intellectual content. ML, MC and CV followed up the patients and provided clinical support. SG performed the statistical analysis. DM, HJ and MTS carried out the laboratory assays and provided technical support. JG and EL performed the drug and metabolite measurements. IF was the research nurse. GP was the pathologist, and carried out the tissue biomarker analysis. FB, FB and AL were the surgeons, and recruited the patients. All authors have read and approved the final manuscript.
